# Treatment with embryonic stem-like cells into osteochondral defects in sheep femoral condyles

**DOI:** 10.1186/s12917-014-0301-9

**Published:** 2014-12-19

**Authors:** Susanna Pilichi, Stefano Rocca, Roy R Pool, Maria Dattena, Gerolamo Masala, Laura Mara, Daniela Sanna, Sara Casu, Maria L Manunta, Andrea Manunta, Eraldo Sanna Passino

**Affiliations:** Department of Animal Science, Agricultural Research Agency of Sardinia, Olmedo, Sassari, 07040 Italy; Department of Veterinary Medicine, via Vienna, Sassari, 07100 Italy; Department of Veterinary Pathobiology, College of Veterinary Medicine and Biomedical Sciences, Texas A&M University, College Station, 77843-4467 TX USA; Department of Surgery, Microsurgery and Medicine, University of Sassari, viale San Pietro, Sassari, 07100 Italy

**Keywords:** Articular cartilage, Embryonic stem-like cell, Fluorescent in situ hybridization, Osteochondral defect, Sheep

## Abstract

**Background:**

Articular cartilage has poor intrinsic capacity for regeneration because of its avascularity and very slow cellular turnover. Defects deriving from trauma or joint disease tend to be repaired with fibrocartilage rather than hyaline cartilage. Consequent degenerative processes are related to the width and depth of the defect. Since mesenchymal stem cells (MSCs) deriving from patients affected by osteoarthritis have a lower proliferative and chondrogenic activity, the systemic or local delivery of heterologous cells may enhance regeneration or inhibit the progressive loss of joint tissue. Embryonic stem cells (ESCs) are very promising, since they can self-renew for prolonged periods without differentiation and can differentiate into tissues from all the 3 germ layers. To date only a few experiments have used ESCs for the study of the cartilage regeneration in animal models and most of them used laboratory animals. Sheep, due to their anatomical, physiological and immunological similarity to humans, represent a valid model for translational studies. This experiment aimed to evaluate if the local delivery of male sheep embryonic stem-like (ES-like) cells into osteochondral defects in the femoral condyles of adult sheep can enhance the regeneration of articular cartilage. Twenty-two ewes were divided into 5 groups (1, 2, 6, 12 and 24 months after surgery). Newly formed tissue was evaluated by macroscopic, histological, immunohistochemical (collagen type II) and fluorescent in situ hybridization (FISH) assays.

**Results:**

Regenerated tissue was ultimately evaluated on 17 sheep. Samples engrafted with ES-like cells had significantly better histologic evidence of regeneration with respect to empty defects, used as controls, at all time periods.

**Conclusions:**

Histological assessments demonstrated that the local delivery of ES-like cells into osteochondral defects in sheep femoral condyles enhances the regeneration of the articular hyaline cartilage, without signs of immune rejection or teratoma for 24 months after engraftment.

**Electronic supplementary material:**

The online version of this article (doi:10.1186/s12917-014-0301-9) contains supplementary material, which is available to authorized users.

## Background

Articular cartilage has a poor intrinsic capacity for regeneration because of its avascularity and very slow turnover both at the cellular and molecular levels. As consequence, defects occurring as a result of trauma or joint disease tend to be repaired with fibrocartilage rather than hyaline cartilage. With time, degenerative processes frequently occur in the regenerated tissue [1-3], which may stabilize or progress in relation to 2 main factors: the width and depth of the defect. It has been demonstrated that sheep articular cartilage osteochondral defects 3 mm wide or less re-fill with normal hyaline cartilage, whereas wider defects are replaced by fibrocartilage which, eventually, degenerates into fibrous tissue [4]. This inferior regenerated tissue is not capable of withstanding the mechanical loads exerted on the tissue during locomotion and the result is eburnation of the subchondral bone [4,5]. In relation to the depth, superficial defects involving only the articular cartilage do not heal spontaneously [1,2,6], while osteochondral defects, penetrating the subchondral bone and thus, gaining access to the mesenchymal stem cells (MSCs) that reside in the bone marrow space, can give rise to regenerated cartilage [7-9].

Surgical treatments used to stimulate cartilage regeneration, in most cases, result only in a delay in the onset of degenerative processes [10-15]. Thus, there is a search for alternative solutions, and cell engraftment is among the most advanced new technologies in cartilage regeneration [16-19]. Considering that in certain degenerative diseases, autologous stem cells are depleted and have reduced proliferative capacity and chondrogenic ability [20,21], the delivery of heterologous cells may enhance regeneration or inhibit the progressive loss of joint tissue [20,21]. Among the several factors to be considered in the choice of the type of cells, there are the ease of harvest, the cell yield and purity and their proliferative and chondrogenic capacity [22]. Autologous chondrocyte implantation (ACI), the first technique used to repair focal cartilage defects [23-25], is associated with donor site morbidity, loss of chondrocyte phenotype upon ex vivo expansion and inferior fibrocartilage formation at the defect site [26-28]. Thus, new extracorporeal cell sources are sought, mainly stem cells. Among them, MSCs [17,18,21,29,30] have the advantages of their immunoevasivity [31] and immunosuppressive effect [32,33], but they have a limited capacity for self-renewal and proliferation, and differentiation potential decreases with increasing donor age [34]. On the contrary, embryonic stem cells (ESCs) are able to self-renew for prolonged periods without differentiation and, most importantly, to differentiate into a large variety of tissues derived from all 3 germ layers [35-38]. Despite the fact that their use is complicated by immunologic incompatibility and possible development of teratomas [39-41], as well as the existence of ethical problems in human application, in the future, the establishment of ESCs lines from ungulates could allow their employment in bulk for basic research and for producing animal models of human diseases [42]. Indeed, the histological appearance of the articular regenerated tissue is essential for the validation of therapeutic interventions [9] and it is likely to be predictive of its functionality and durability [43]. Since in humans it is possible to perform only small arthroscopic sampling to evaluate the histological aspect of the regenerated tissue, deep histological studies can be performed in vivo only in animal models. To date few studies concerning cartilage regeneration have been performed in animal models in vivo [44-52] and, until recently, most of the research on stem cells has been carried out in small laboratory animals [30,53-55]. However, these species do not represent an optimal model for achieving cartilage regeneration in human. On the contrary, the larger size and weight of adult sheep, which place greater weight-bearing loads on the healing site, as well as the structural, biochemical, physiological and immunological similarities to man and the ease and low cost of their management with respect to other species, make sheep an optimal experimental model for future clinical applications in humans [50,56].

The stem origin of the in situ differentiated cells in the regenerated tissue was confirmed by the FISH technique.

This experiment aims to evaluate if the treatment with ES-like cells into osteochondral defects in the medial femoral condyles of adult sheep can enhance the regeneration process of articular cartilage over a long time period, without formation of teratomas.

## Results

Only 17 animals were included in the statistical analysis, because 5 sheep died from toxaemic gastroenteritis after breaking through paddock fencing and freely consuming grass covered with frost. One sheep belonging to the 24 month group died at about 12 months post-surgery, and hence was included with the 12 month group. Analysis was thus performed on 2 sheep in the 1 month group, 3 sheep each in the 2 and 6 month groups, 5 sheep in the 12 month group and 4 sheep in the 24 month group.

### Embryo sexing and ES-like cell characterization

**Figure 1:**
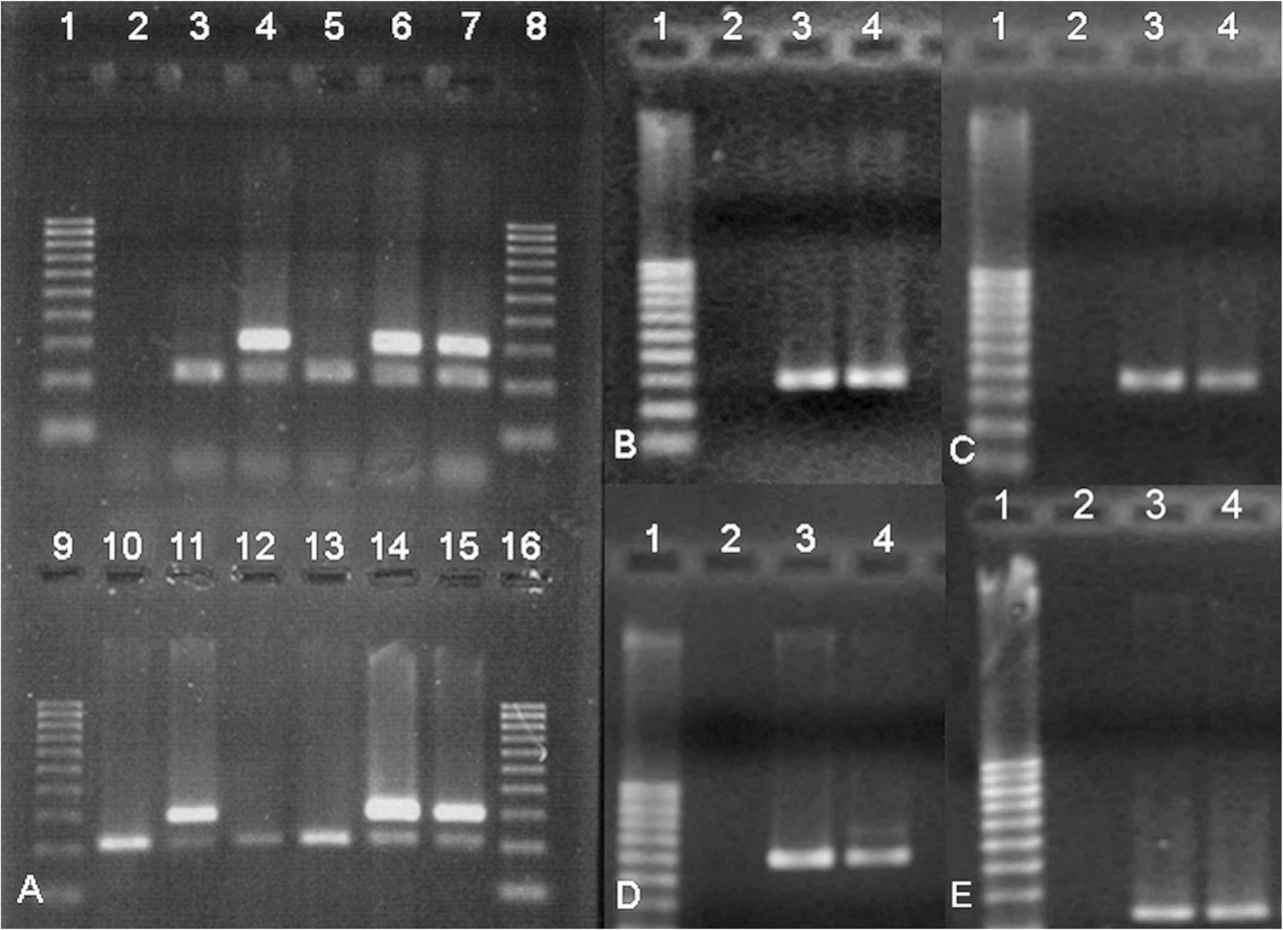
**Embryo sex determination and ES-like colonies gene expression** ***.*** **A)**: Embryo sex determination. Lanes 1, 8, 9, 16: DNA marker 100–1000 bp ladder (Sigma); lane 2: negative control (no DNA); lane 3: female positive control (sheep oviductal cells); lane 4: male positive control (male lamb fibroblasts); lanes 5, 10, 12, 13: female embryos; lanes 6, 7, 11, 14, 15: male embryos. **B,C, D, E)** ES-like colonies gene expression. **B)** *Oct4*gene expression; **C)** *Sox2* gene expression; **D)** *Nanog* gene expression; **E)** *Stat3* gene expression. Lane 1: DNA Marker 100–1000 bp ladder (Sigma); lane 2: negative control (no cDNA); lane 3: in vitro produced sheep blastocyst, used as positive control; lane 4: ES-like cells.

Sexing PCR allowed selection of male embryos for production of male ES-like cells. Male embryos showed 2 bands corresponding to the sex-determining region Y-linked gene (SRY) and the sheep SAT 1,114 DNA repeat unit (SAT) sequences, while female embryos showed only the band corresponding to the autosomal sequence SAT (Figure 1A). ES-like colonies demonstrated their staminality state by positive immunostaining for SSEAs monoclonal antibodies (mAbs) and positive gene expression for *Oct 4*, *Nanog*, *Sox 2* and *Stat 3* genes (Figure 1B, C, D, E), and their undifferentiated state by the absence of staining with any of the anti-cytokeratin-18, Fe-C6, F1-652 and FORSE-1 mAbs.

### Clinical assessment

Eight sheep were moderately lame in both hind limbs the day after surgery, but they all walked normally by day 9. No further problems with locomotion were noted in any of the animals during the remainder of the study.

### Macroscopic assessment

Since the interaction between time and treatment was not significant for any of the analysed variables, it was removed from the statistical model, to test the effect of the main factors (treatment and time period). No significant effect of treatment was observed in the macroscopic assessment, either in the total or in the single category scores throughout all considered time periods (from 1 to 24 months) (Table 1). However, ES samples showed higher least square means with respect to empty defects (ED) samples, both in the total macroscopic score, in the surface appearance and in the filling of the defect, while the integration of edges was slightly better in the ED animals (Table 1). For sake of clarity in the presentation of results, the interaction was maintained in the statistical model in order to estimate the least square means for each period per treatment level (Additional file 1: Table S3).

**Table 1:** Least square means ± standard error of ES and ED macroscopic scores compared from 1 to 24 months (17 ESM and 17 ED)

**Categories**		**Treatments**	
	**ES** ^*^	**ED** ^**†**^	**P value**
Total macroscopic score	4.66 ± 0.4	3.81 ± 0.4	n.s.^‡^
Surface appearance	1.51 ± 0.2	1.06 ± 0.2	n.s.
Filling of defect	2.09 ± 0.2	1.65 ± 0.2	n.s.
Edges integration	1.06 ± 0.2	1.10 ± 0.2	n.s.

^*^Embryonic stem-like cells engrafted in the medial femoral condyle; ^**†**^Empty defect; ^‡^not significant.

### Evaluation at 1 month

**Figure 2:**
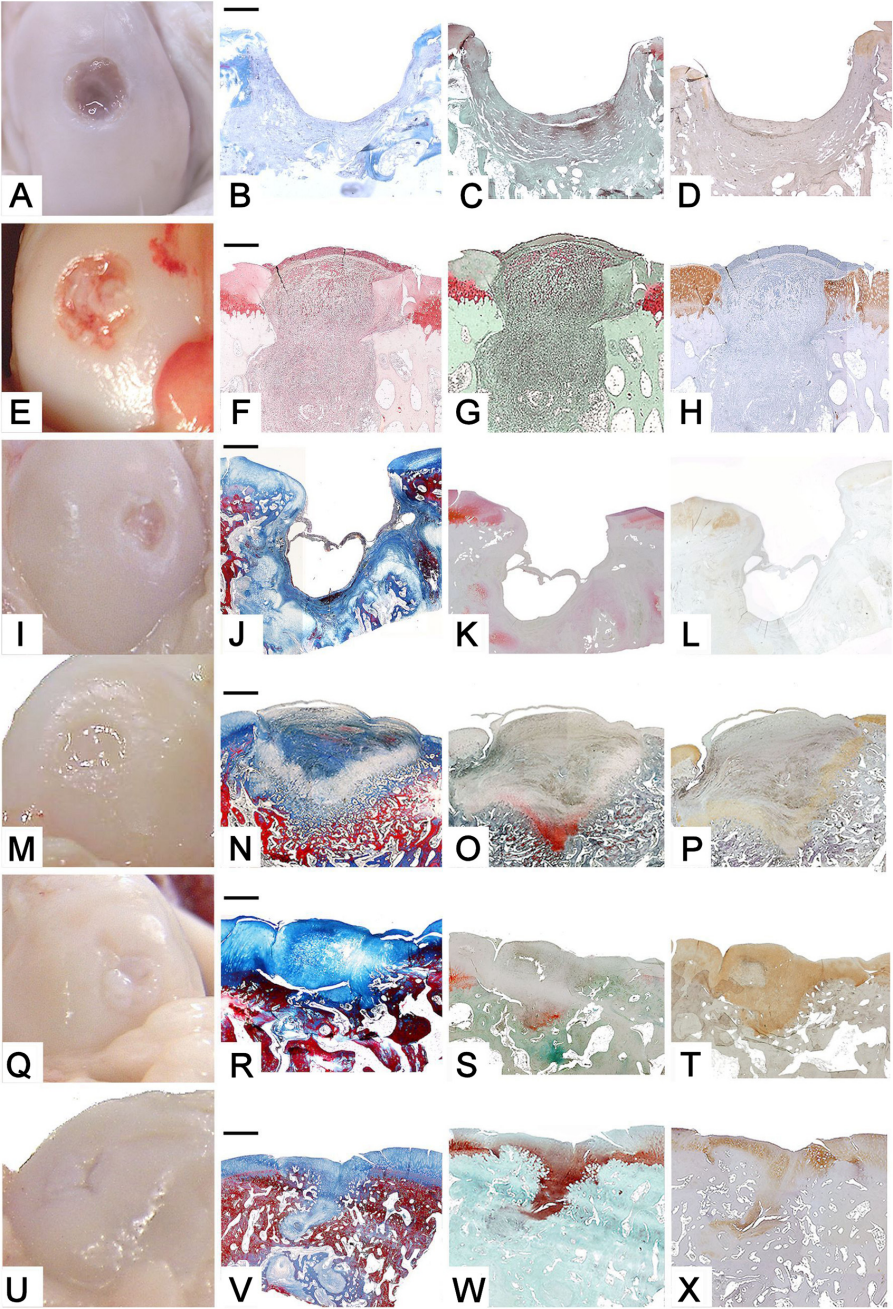
**Femur, sheep.** Embryonic stem-like cells engraftment in the medial femoral condyle (ES) and empty defects (ED) at 1, 2 and 6 months from surgery. **A-D)** ED at 1 month from surgery. **E-H)** ES at 1 months from surgery. **I-L)** ED at 2 months from surgery. **M-P)** ES at 2 months from surgery. **Q-T)** ED at 6 months from surgery. **U-X)** ES at 6 months from surgery. **A-E-I-M-Q-U)** macroscopic appearance; **B-J-N-R-V)** histological sections, Azan-Mallory staining. 2X magnification; bar: 1 mm. **F)** Haematoxylin-eosin staining, **C-G-K-O-S-W)** Safranine-O staining. **D-H-L-P-T-X)** Collagen type II immunostaining.

The total macroscopic score showed better healing in the ES samples than in the ED. The ES samples had a better surface appearance and had greater filling with a soft, red tissue (Figure 2A) than the ED samples (Table 1), where only the bottom and the sides of the defect were covered by the proliferating tissue (Figure 2E). Edge integration was absent in all samples.

### Evaluation at 2 months

The overall healing process decreased in the ES samples with respect to the control group. Surface appearance showed a flat whitish tissue with a regular and smooth surface which completely filled the ES (Figure 2O), while it only partially filled the ED (Figure 2I), despite the fact that both treated and control groups showed the same least square mean in both categories. In both samples the newly proliferating tissue still lacked continuity with the host cartilage, though the ED group showed a better least square mean (Table 1).

### Evaluation at 6 months

Both samples had opalescent-appearing regenerated tissue with extended superficial cracks (Figure 2S and W), showing the same least square means in the total macroscopic score and in the filling of the defect. ES samples had slightly higher scores for surface appearance, and slightly lower scores for edge integration (Table 1).

### Evaluation at 12 months

**Figure 3:**
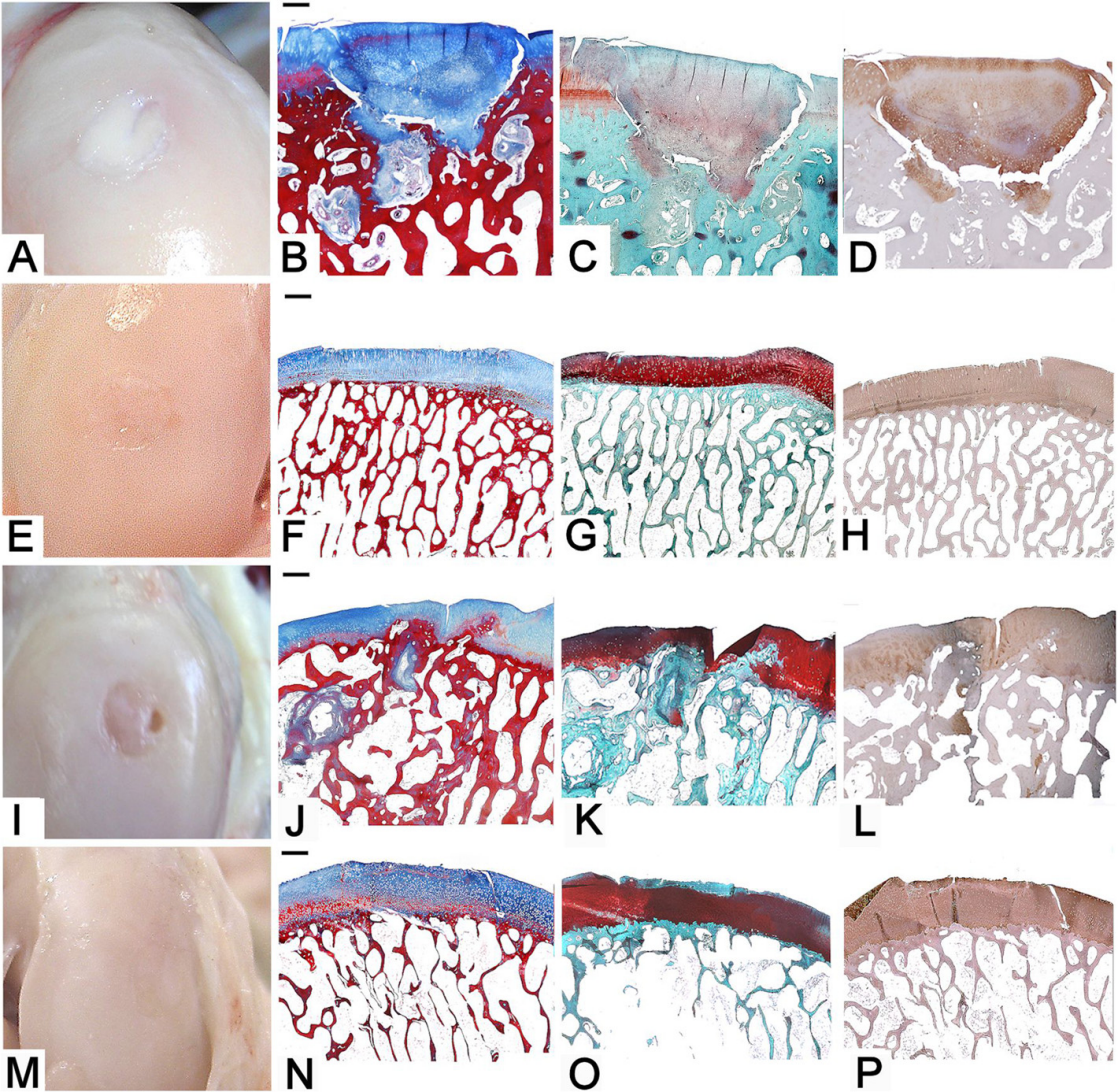
**Femur, sheep.** Embryonic stem-like cells engraftment in the medial femoral condyle (ES) and empty defects (ED) at 12 and 24 months from surgery. **A-D)** ED at 12 months from surgery. **E-H)** ES at 12 months from surgery. **I-L)** ED at 24 months from surgery. **M-P)** ES at 24 months from surgery. **A-E-I-M)** macroscopic aspect; **B-F-J-N)** histological sections, Azan-Mallory staining, 2X magnification; bar: 1 mm. **C-G-K-O)** Safranine-O staining. **D-H-L-P)** Collagen type II immunostaining.

Regenerated tissue, which appeared similar to pre-existing cartilage, completely filled both defects, and was continuous with the host tissue (Figure 3A and E). All examined categories showed higher least square means in ES samples with respect to the control group (Table 1).

### Evaluation at 24 months

Both defects were completely filled by a newly formed tissue, smooth and white like hyaline cartilage. Edges were totally integrated with the host tissue, with the exception of one ED which showed a small fissure near the edge (Figure 3I and O). Once again, all examined categories showed higher least square means in ES samples with respect to the control group, and a slight overall decrease with respect to the 12 month time period (Table 1).

### Histological assessment

As for the macroscopic assessment, the interaction between time and treatment was not significant for any of the histological variables and was removed from the model to test the effect of the main factors (treatment and time). ES performed significantly better in the total histological score (p < 0.001) and in the filling of defect, cartilage, matrix, bone and edge categories (p < 0.05) with respect to ED, while no significant differences were observed between treated and control groups in degeneration or vascularity (Table 2), though higher least square means were reached by ES samples than control (Table 2). Once again, to better clarify the evolution of the regeneration process, the least square means for each period and treatment were calculated (Additional file 1: Table S4).

**Table 2:** **Least square means ± standard error of histological scores compared throughout all considered periods**

**Categories**	**Treatments**		**P value**
	**ES** ^*^	**ED** ^**†**^	
total histological score	36.33 ± 1.1	30.41 ± 1.1	< 0.001
filling of defect	1.65 ± 0.1	1.22 ± 0.1	< 0.05
cartilage	6.75 ± 0.4	5.20 ± 0.4	< 0.05
matrix	5.83 ± 0.4	4.46 ± 0.4	< 0.05
bone	4.57 ± 0.2	3.90 ± 0.2	< 0.05
edges	1.81 ± 0.2	1.13 ± 0.2	< 0.05
degeneration	10.86 ± 0.2	10.61 ± 0.2	n.s.^‡^
vascularity	4.86 ± 0.5	3.89 ± 0.5	n.s.^‡^

^*^Embryonic stem-like cells engrafted in the left medial femoral condyle; ^†^Empty defect created in the right medial femoral condyle. ^‡^not significant.

### Evaluation at 1 month

Proliferating granulation tissue rich in vessels and fibroblasts completely filled the cavity in the ES samples, while it covered the bottom and the walls only of the ED samples. Foci of endochondral ossification were detected at the bottom of the ES samples; there was no integration between new and host tissue, despite their close proximity. Regenerated tissue was negative for proteoglycans and collagen type II production in both groups (Figure 2B, C, D and F, G, H). Higher least square means were reached by ES samples with respect to controls in the total histological score, filling of defect, cartilage, matrix and degeneration categories, while they were equal in both treated and control groups in bone and edges categories (Additional file 1: Table S4).

### Evaluation at 2 months

ES were superficially filled by fibrous tissue and by subchondral ossification at the bottom, while ED were less completely filled and one sample showed a deep hole covered by a thin bridge formed by the tangential layer. Integration between new and host tissues was present only at one edge in the ES. An initial production of proteoglycans and a mild staining for collagen type II were detectable at the bottom of both defects, while the fibrous tissue in the top was negative (Figure 2L, M, N and P, Q, R). Higher least square means were reached by ES samples with respect to controls in all the histological categories (Additional file 1: Table S4).

### Evaluation at 6 months

Both samples contained fibrocartilage in the superficial part. There was woven bone with foci of subchondral ossification at the bottom of the ED samples. Cartilage clefts and small subchondral cysts were sometimes present. Clefts were deeper and localized at the edges with surrounding degenerated matrix in the ED, in which continuity between tangential layer proliferation and subchondral ossification was sometimes interrupted. Proteoglycans were conspicuous in the ES, especially in the deeper part of fibrocartilage, while an initial production was detected within the foci of subchondral ossification in the ED. A marked staining for collagen type II was present in the regenerated tissue of both samples, together with some negative areas in the middle and deeper parts (Figure 2T, U, V and X, Y, Z). Higher least square means were reached by ES samples with respect to controls in the total histological score, matrix, bone and edges categories, while they were equal in both ES and ED groups in the filling of defect category, and slightly lower in ES in respect to ED in the cartilage and degeneration categories (Additional file 1: Table S4).

### Evaluation at 12 months

ES samples showed immature hyaline cartilage and lamellar bone, and the ED group had immature hyaline cartilage spaced out by fibrocartilage and woven bone, where continuity between tangential layer proliferation and subchondral ossification was often absent, and only one edge was integrated. ED samples sometimes showed deep cracks involving the whole depth of the cartilage. Proteoglycan staining was strong in the ES group and minimal in the ED, while collagen type II immunostaining was intense in both groups, with some negative areas in the centre of the regenerated cartilage in the ED animals (Figure 3B, C, D and F, G, H). Higher least square means were reached by ES samples with respect to controls in all the histological categories (Additional file 1: Table S4).

### Evaluation at 24 months

**Figure 4:**
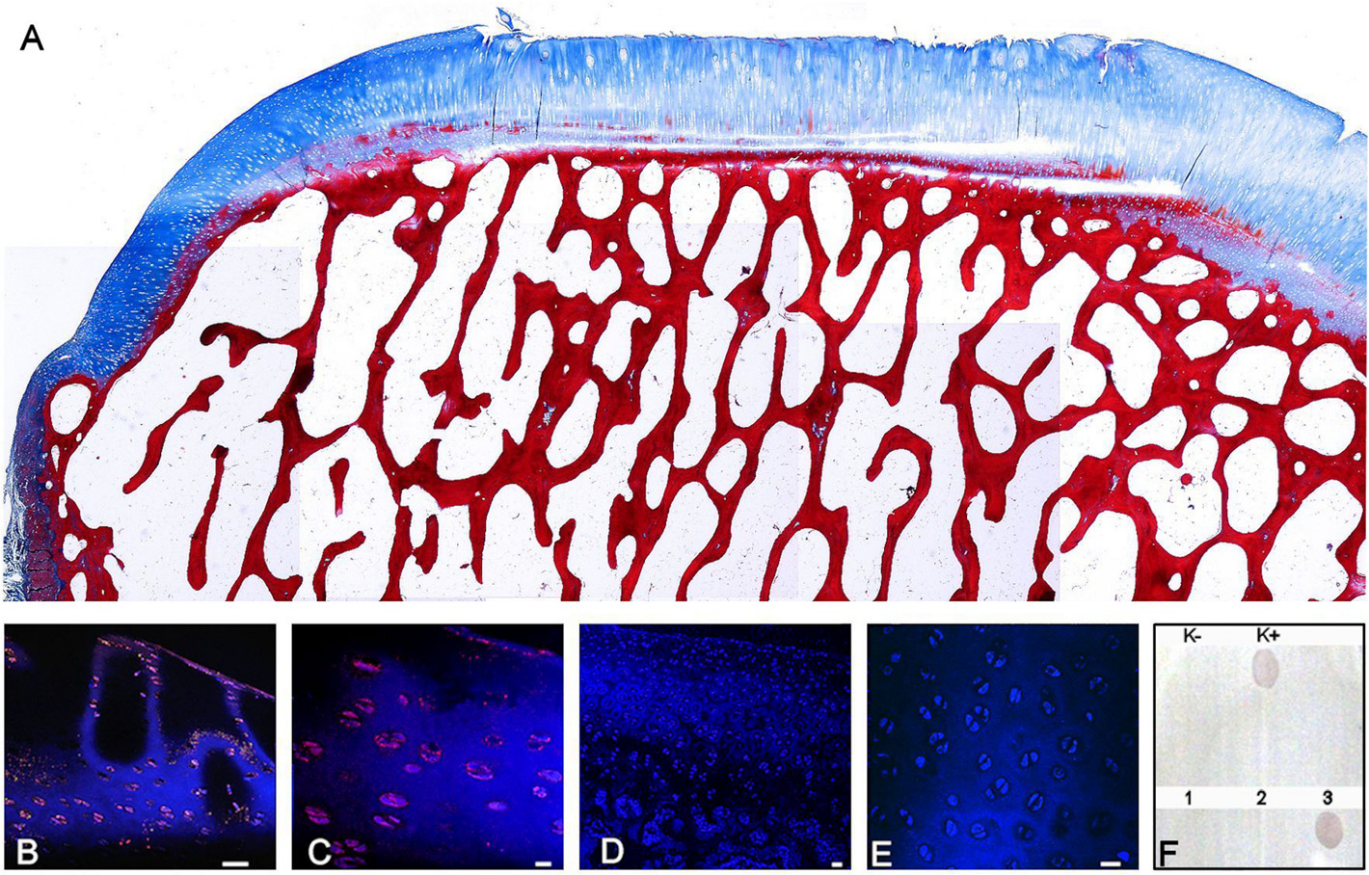
**Femur, sheep.** Embryonic Stem-like cells engraftment at 24 months from surgery. **A)** Histological aspect: Azan-Mallory staining; 2× magnification; bar: 500 μm. **B)** Fluorescent in situ hybridization (FISH): positive signals in chondrocytes derived from ES-like cells. 40× magnification; bar: 50 μm. **C)** Same field, 60× magnification; bar: 10 μm. **D)** Normal female adult articular cartilage from the right lateral femoral condyle (negative control). No signals are detected within chondrocytes. 20× magnification; bar: 30 μm. **E)** Same field, 40× magnification; bar: 20 μm. **F)** Dot-Blot test with SRY probe. K-: spot female sheep fibroblasts (negative control), K+: spot male sheep fibroblasts (positive control), 1–2: spot female ES-like cells, 3: spot male ES-like cells.

The appearance of the newly formed tissue in ES animals was comparable to mature hyaline cartilage, lying on a well-reconstituted subchondral plate formed by lamellar bone; integration between regenerated and host tissue was present at both edges and mild to moderate articular deterioration was seen (Figure 3P). The healed cartilage consisted of a superficial zone with proliferating germinal cells and collagen fibres distributed parallel to the articular surface, overwhelming an area rich in chondrocytes organized in large clones, and a transitional zone characterized by palisading chondrocytes and spring-shaped arrangements of collagen fibres. Subjacent to this was a radial zone with hypertrophic chondrocytes and collagen fibres arranged perpendicular to the articular surface, and a deep zone with calcification and formation of tidemark, still discontinuous (Figure 3P and Figure 4A). On the contrary, ED were filled by immature hyaline cartilage in the upper part and woven bone at the bottom, where moderate subchondral cysts were sometimes detected (Figure 3L). Intense proteoglycan and collagen type II staining was detected in the newly formed cartilage (Figure 3M, N and Q, R). Higher least square means were reached by ES samples with respect to the control group in all the histological categories (Additional file 1: Table S4).

### FISH detection

Dot-Blot showed positive spots with both DNA derived from ES-like cell samples and male fibroblasts (positive control), whereas female fibroblasts DNA (negative control) was negative, confirming the specificity of the chosen probe (Figure 4A). FISH showed positive intranuclear signals only in the ES-like derived cells found in the newly formed tissue at all-time points, while controls were always negative (Figure 4B, C, D, E).

## Discussion

In this study ES showed significantly better histological evidence of healing when compared to ED in most of the examined categories. To our knowledge, this is the first time that ES-like cells have been engrafted in sheep and then evaluated out to 24 months post-surgery. Previously, engraftments had been assessed at a maximum period of 18 months in sheep [4], 12 months in goats [21], 8 months in horses [18] and 6 months in laboratory animals [30,53-55].

Sheep have been shown to be a valid model for human translational research when compared with laboratory animals, due to the increased stifle size, less effective native cartilage regeneration and longer lifespan. Moreover, a recent study comparing the differences in geometry and biomechanical properties of human, porcine, bovine and ovine articular cartilage, found that human cartilage had a significantly larger flexibility with respect to porcine and bovine cartilage, but not to ovine cartilage [57]. In addition, from the regulatory point of view, the ovine model is one of the suggested large animal models for pre-clinical studies [48]. However, in previous studies in small ruminants (sheep [4,13,14,49,52] and goats [5,7,21]), most animals were euthanized at 6 months, paralleling the time frame of experiments in laboratory animals. According to the authors, this is insufficient time to observe complete regeneration in large animals. Schneider-Wald [58] states that the follow-up period for assessment of the effectiveness of cartilage regeneration is 12 months. Moreover, in sheep, several authors [46,47,49-51] worked with chondral defects, despite the fact that this type of defect can’t heal spontaneously [1,2,6]. Most of the experiments performed in sheep employed surgical techniques only without the use of cells [4,13,52,59] and, even when stem cells were used, the methods of delivery to the cartilage varied from direct injection to implantation with a large variety of scaffolds [60]. For these reasons, it is difficult to compare results obtained in this experiment with previously reported trials.

Sexing PCR performed on trophoblastic cells lysed during the immunosurgery procedure confirmed that the choice of the sex-determining region Y-linked gene sequence (SRY) as a marker for ES-like cells was effective and reliable for this purpose [61]. To detect the stem origin of differentiated cells, several authors [21,49,62] employ green fluorescent protein (GFP) transduced cells. However, this procedure is applicable only on cells that can be expanded ex-vivo in large amounts, such as MSCs [21,49] and rodent ESCs [62]. On the contrary, ungulate embryonic stem cells show an early in vitro differentiation [42,63], which does not allow production of the needed amount of cells for the application of this technique. To overcome this problem, the authors engrafted only male ES-like cells in female sheep, and the SRY sequence was used as a marker for the FISH technique, which allowed to confirm the stem origin of differentiated cells.

ES-like colonies were positive for the SSEAs mAbs and the gene expression for *Oct 4*, *Nanog*, *Sox 2* and *Stat 3* genes, and negative for any of the mAbs marking the differentiated state, demonstrating that the engrafted cells were stem cells, in agreement with others [64-76]. The low number of ES-like cells engrafted was due to the early in vitro differentiation of sheep ES-like cells, as mentioned above [42,63] which does not provide the higher numbers of cells used in other studies [18,30,62]. This forced us to use cells derived from the first passage, when they were still in an undifferentiated state. The establishment of ES cell lines of domesticated ungulates is of interest both for basic research, such as the study of comparative embryology and the comprehension of the mechanisms of cell biology related to stem cell maintenance and differentiation [42], and applied research, such as the creation of models of human genetic diseases and cell transplantation therapy [42].

The lateral para-patellar approach was shown to be reproducible in a standardized fashion and reliable for long-term success of the cell therapy. Indeed, all animals walked normally 9 days after surgery and no signs of major wound infection or limited range of motion were detected, similar to previous reports in sheep [4,13,52], goats [5,21] and rabbits [30,55] . Medial patellar dislocation was necessary to reach the medial femoral condyle. Lateral access guarantees greater resistance of the sutures in the various layers of the closure, thanks to the presence of the fascia lata. Arthroscopy was not used because it did not allow the deposition of the cell-fibrin graft in the defect. The surgeons noted that this technique was quite invasive and could have negatively influenced the results, due to the loss of synovial fluid during the surgical procedure and consequent dehydration of articular cartilage. These problems have been overcome in later studies (data not shown) with the introduction of the mini-arthrotomy technique. Particular attention has been paid to meticulously removing all calcified cartilage between the tidemark and the osteochondral junction, according to Hurtig [77]. This cartilage layer appears to be an effective barrier to the invasion of the blood and precursor cells from the subchondral bone, resulting in a lack of attachment of the regenerated tissue to subchondral bone [77].

No significant macroscopic differences were observed between ES and ED samples. This is probably because of the absence of the assessment of transverse sections, perpendicular to the articular surface and about 1–2 cm deep, which could have allowed evaluation of the quality of the newly formed tissue deep in the samples, together with the detection of subchondral cysts. In any case, this experiment demonstrates that, in sheep, tissue resembling cartilage appears only at 6 months post-surgery, as noted by other authors [4,78], and integration of edges is complete at 12 months, as reported by Akens [4] but in contrast to the findings of Schleicher [78]. It is interesting to note that at 24 months there was a slight overall decrease in the regeneration process in both groups (more marked in the ED group) as compared to 12 months.

The ES group performed significantly better in most categories of histological assessment with respect to ED. Beginning at the earliest time points, this group had more normal architecture and a better quality of matrix in the newly formed cartilage, similar to the findings of Akens [4]. These findings suggest the enhancement of the physiological regeneration process exerted by ES-like cells, probably by means of paracrine signals [1,79]. Several authors [8,17,19,80-83] outline the complex interplay existing between proteoglycans and collagen type II, the two most important components of the hyaline cartilage matrix: the collagen fibrils serve to anchor the proteoglycan matrix and contribute to resisting extrinsic forces during loading and the intrinsic swelling that occurs within the proteoglycan domain, while proteoglycans are responsible for the generation of a hydrostatic pressure within cartilage matrix, which allows it to counteract the loads transmitted to it from the long bones during normal joint articulation.

Tidemark appeared at 12 months after surgery, without statistically significant differences between treatment groups. It was still discontinuous in most of samples at 24 months post surgery, suggesting that the regenerative process was not yet complete. The presence of the tidemark is an important finding: it has been reported that this calcification front is in a state of dynamic equilibrium, where factors promoting mineralization are probably counterbalanced by substances that inhibit or limit the extent of calcification, and this process of active calcification and subsequent endochondral ossification seems to be integral to the shape of the joint and, therefore, to the distribution of load [84].

In relation to bone remodeling, lamellar bone was detected 12 months after surgery in most of the ES samples, while ED controls showed mostly woven bone. This last finding is in contrast with Jackson [7] who, in empty defects in goats at 13 months, found sclerotic bone surrounding the defect adjacent to subchondral areas, sparse bone resorption and marked endochondral bone formation, probably because of the difference in the anatomy and ambulation between sheep and goats. These results further confirm the benefits that occurred in the defects treated with ES-like engraftments, considering that a good remodeling of subchondral bone provides the right biomechanical support for the hydrostatic compression in the cartilage, which is of critical importance for the differentiation of hyaline cartilage [8,81].

ES samples showed very good integration between the newly formed cartilage and the host tissue as compared to ED samples, particularly evident at 12 and 24 months. These findings, similar to those reported by Akens [4], seem likely to be predictive of the functionality and durability of the regenerated tissue. It is well known that the gap between implant and host tissue is responsible for the initiation of cartilage degeneration, as a result of a penetration of the synovial fluid into the host-graft interface which results in resorption within the subchondral bone area and cyst formation [4,7,59].

Mild to moderate signs of articular deterioration (delamination, fibrillation or clefts of the cartilage surface) predominated in the ED samples. Subchondral bone cysts, varying from mild to severe, were detected in both groups, with a higher incidence at 6 months, similar to previously published reports [4,7,59]. This could be due to the particular texture of the regenerated tissue at this time point, enough hard to allow the penetrating synovial fluid to reach high pressures but, at the same time, too soft to counteract it, with consequent collapse of the walls and enhancing of the cyst formation.

No signs of immune rejection or teratoma occurred in any of the ES-like engraftments, despite the fact that engrafted animals were not immunosuppressed. It could be due to the osteochondral microenvironment, which may have forced the implanted cells to differentiate toward the osteogenic and chondrogenic lineage [39-41,85] or to the small number of cells implanted [16]. A recent study [62] hypothesizes that cycling loading may affect the differentiation of stem cells in osteochondral defects to the osteocyte and chondrocyte phenotypes.

## Conclusions

Heterologous embryonic stem-like cells implanted into osteochondral defects in sheep femoral condyles enhanced the regeneration of the articular hyaline cartilage, without the occurrence of immune rejection or teratoma formation even at 24 months post-surgery. These findings are encouraging for safe translation to human clinical trials.

## Methods

### Ethics statement

The animals were maintained and treated in accordance with Legislative Decree 116/92 guidelines based on European Directive 86/609/EEC. Animal welfare was routinely checked by veterinarians from VTH (Veterinary Teaching Hospital) of the Department of Veterinary Medicine and every effort was made to minimize the number of animals used and their suffering (see ARRIVE checklist in Additional file 2). The experimental protocols were approved by the Ethical Committee of the University of Sassari and by the veterinary control officers of animal protection in experimental and clinical studies conducted at the University of Sassari (Auth. of 06/06/2012).

If not indicated otherwise, all chemicals were purchased from Sigma-Aldrich.

### Experimental design

Twenty-two Sarda ewes, about 5.5 years old and weighing approximately 45 kg, without muscular-skeletal pathologies, were used in this experiment. Sheep were divided into 5 groups which were euthanized at 1, 2, 6, 12 and 24 month post-surgery, respectively. Each group was composed of 4 sheep with the exception of the 24 month group, where 6 sheep were allotted to account for possible deaths during the study period. ES-like cells were engrafted in the osteochondral defect created in the left medial femoral condyle (ES), while the identical defect created in the controlateral stifle joint was left untreated (ED) and served as a control. Thus, a total of 44 defects were created: 22 ES and 22 ED (Table 3).

**Table 3:** **Experimental design**

**Period-groups**	**N° of sheep**	**Stifle joint (medial femoral condyle)**	**Treatment**
1 month	4	Left	ES^*^
		Right	ED^**†**^
2 months	4	Left	ES
		Right	ED
6 months	4	Left	ES
		Right	ED
12 months	4	Left	ES
		Right	ED
24 months	6	Left	ES
		Right	ED
	22		44

^*^ES-like engraftment in the medial femoral condyle defect; ^**†**^Empty Defect.

### In vitro embryos production, embryo sexing, ES-like cell production and characterization

About 100 embryos at the blastocyst stage were produced following in vitro maturation, fertilization, culture and vitrification procedures, as previously described [86]. Since the presence of the SRY sequence was used as a marker to detect male ES-like cells in the regenerated tissue, only male embryos were selected to produce ES-like cells, by means of a duplex PCR [61]: the 2 pairs of primers (Life Technologies) identified both the SRY gene sequence of male embryos (SRY-fwd 5′-CTCAGCAAAGCACACCAGAC-3′, SRY-rev-5′-GAACTTTCAAGCAGCTGAGGC-3′), and the SAT sequence in both sexes (SAT-fwd 5′-TGGAAGCAAAGAACCCCGCT-3′, SAT-rev 5′-TCGTGAGAAACCGCACACTG-3′). SAT was used as positive control for the reaction. Male lamb fibroblasts and sheep oviductal cells were used as positive and negative controls, respectively. Amplification products were analysed on 2% agarose gel stained with ethidium bromide and observed under ultraviolet light.

The inner cell mass (ICM) from each numerically identified male embryo was isolated by immunosurgical complement mediated lysis of trophoblastic cells and separately cultured on a mouse fibroblast feeder layer for 5 to 6 days [65], to obtain ES-like colonies. These were characterized both immunocytochemically, by detection of surface antigens for staminality SSEA-1, SSEA-3 and SSEA-4 (Developmental Studies Hybridoma Bank - DSHB, University of Iowa, Iowa City, IA) [65] (Table 4) and by assessment of the gene expression of *Oct 4*, *Nanog*, *Sox 2* and *Stat 3* genes [68]. To assess the absence of differentiation towards germinal layers, cytokeratin, early mesoderm, embryonic myosin and neural precursor cell specific primary mAbs (DSHB) were used [65] (Table 4).

**Table 4:** **Antibodies used for immunocytochemistry**

**Antigens**	**Detected tissue**	**I° Antibodies**	**Antibodies dilution**	**II° Antibodies**
SSEA-1	Staminality Marker	Mc 480	1:100	Anti-mouse IgM^c^
SSEA-3	Staminality Marker	Mc 631	1:100	Anti-rat IgM (Pierce Thermo Scientific)
SSEA-4	Staminality Marker	Mc 813-70	1:100	Anti-mouse IgG
Cytokeratin	Endoderm	Anti-cytokeratin peptide 18	1:100	Anti-mouse IgG
Early mesoderm	Early mesoderm	Fe-C6	1:100	Anti-mouse IgM
Embryonic myosin	Late mesoderm	F1-652	1:100	Anti-mouse IgG
Neural precursor cells	Ectoderm	Forse-1	1:100	Anti-mouse IgM

### Preparation of cells for engraftment and surgical procedures

For each graft, 2 to 3 male ES-like colonies were harvested from the Petri dish, disaggregated using trypsin and pooled together, as previously described [16]. About 500,000 cells were embedded in 60 μl of fibrin glue and engrafted into the cartilage defect.

All animals were given a general health examination, dewormed and had their claws inspected prior to the experimental surgery. After sedation (Diazepam 0.4 mg/kg IV) and sacro-coccigeal (S4-Co1) epidural anaesthesia (Lidocaine 2 mg/kg), the induction of the anaesthesia was performed with Ketamine (3 mg/kg IV) and maintained with Ketamine (3 mg kg^−1^ h^−1^) and Fentanyl (8 mcg kg^−1^ h^−1^). A lubricated stomach tube of 1 cm of inner diameter was inserted into the rumen to prevent bloating. The animal was positioned in dorsal recumbency in a cradle for thoracic containment, leaving posterior limbs free. After disinfection and preparation of the surgery field, with the knee placed in maximum flexure, a para-patellar arthrotomy of both stifle joints was performed using a lateral approach and medial patellar dislocation, to expose the articular surface of the weight-bearing area of the medial femoral condyle. A cranio-lateral cutaneous incision, of about 8 cm in length, was performed over the rotula, followed by subcutaneous tissue incision to visualize the septum between the superficial lamina and the fascia lata and the biceps-femoral muscle proximally and the retinaculum distally. Finally, the incision of the articular capsule allowed access to the joint. A full-thickness defect was made in the articular cartilage using a 6 mm diameter punch to mark off the edges of the defect and to cut down into the cartilage until the calcified portion was reached. A curettage was then performed by using a little Williger spoon to remove cartilage debris. To make the defect walls perfectly perpendicular to the articular surface and to remove the whole calcified layer of cartilage, a motorized drill for spinal surgery with a stop-mechanism, so that the depth drilled was exactly the same in all samples, was used. Thus, all defects had the same diameter (6 mm) and depth (2 mm) and involved the subchondral bone (osteochondral defects). During drilling, the area was infused with saline solution to cool the tissue and to avoid dehydration of articular cartilage due to the loss of synovial fluid during the surgical procedure. Finally, an abrasion of the subchondral bone plate was carried out using a 18 G needle, to allow bleeding of the bottom of the defect and the consequent access of both autologous mesenchymal stem cells MSCs and growth factors from the underlying bone marrow. The left joint received the ES-like cells embedded in fibrin glue, while the right knee was left untreated to serve as a control. All animals received an antibiotic and an antinflammatory agent (amoxicillin 25 mg/kg im and ketoprofen 2 mg/kg im) before the patella was repositioned and the wound sutured. All surgical procedures were performed by the same operator, respecting animal welfare laws. Antibiotic and antinflammatory therapy (amoxicillin 40 mg/kg/day im and ketoprofen 2 mg/kg/day im) was administered for 5 days. All animals were kept confined in paddocks for 10 days in groups of 6 animals each, and then were allowed to roam freely on pasture for the rest of the study.

### Gross assessment

Immediately after euthanasia, the defects were photographed to allow the assessment by 2 blinded observers. A semi-quantitative scoring system, developed by the International Cartilage Repair Society (ICRS), (Additional file 1: Table S1) was used and the values obtained were averaged. Observations were initially recorded as percentages (surface appearance , filling of the defect, and graft-host integration at the margins). These percentages were converted to scores from 0–3 for purposes of statistical analysis.

### Histological and immunohistochemistry procedures and evaluation

Samples containing regenerated tissue, adjacent host cartilage and subchondral bone (thickness: 0.5 cm; width: 2–3 cm; height: 1–2 cm; distance of cuts from edges of defect: 0.2 cm) were harvested using a water-cooled circular saw. The tissue blocks were fixed in 10% neutral-buffered formalin and then decalcified with a 1:1 citric acid/formic acid solution for about 10 days. After washing in running tap water for 4–8 h to remove all traces of decalcification solution, samples were routinely processed for paraffin embedding. Three μm sections were mounted on positively charged slides (Superfrost Plus, Gerhard Menzel GmbH) to prevent detachment and stained with hematoxylin-eosin for general tissue evaluation, Azan-Mallory to demonstrate collagen fibres and safranine-O to detect proteoglycans. All slides were processed together to avoid variability. Immunohistochemistry was used to detect collagen type II in the cartilage matrix. After deparaffinization, re-hydration, protease K and Endo- and Tissue-Blocker (Biomeda) treatments, slide were incubated overnight at 4°C with a primary antibody (anti-Collagen type II CIICI - DSHB) at 1:200 dilution, followed by the secondary biotinylated antibody (Biomeda) for 10 min at room temperature (R.T.), and by 3% peroxidase solution for 10 min at RT. After staining with diaminobenzidine (Biomeda) and counterstaining with haematoxylin, slides were dehydrated, coverslipped, observed under light microscopy and digital images were captured. The lateral femoral condyle was used as positive (articular cartilage) and negative (subchondral bone) controls.

Two independent observers evaluated the regeneration progress using a grading system developed by the authors and derived from Caplan [87] (Additional file 1: Table S2) and the 2 values obtained for each sample were averaged. A total score of 5 indicated the worst possible healing, while 56 indicated the best.

### FISH technique

FISH assay was performed on one sample from each period, as previously described [88], using the DNA probe 50 bp in length (biotin-5′AAAGGGAGGGAGAGACCAAAGAAGTAGATGATGATGATGATGAAGTGATC 3′) built on the SRY sequence. To assess the effectiveness of the probe, a membrane Dot-Blot test had been previously performed on the ES-like cell samples, using male and female fibroblasts as positive and negative controls, respectively. Sections mounted on positively charged slides (Superfrost Plus, Gerhard Menzel GmbH), were deparaffinised, rehydrated, permeabilized by 0.8% pepsin treatment at 37°C for 30 min, post-fixed and incubated with a prehybridization solution (50% hybridization solution, 43% formamide and 7% double-distilled water) at 50°C for 2 h. The probe (2000 ng/μl) was denatured at 98°C for 10 min and immediately placed on ice. Tissue was denaturated at 98°C for 8 min with 200 μl of hybridization solution (50% hybridization solution, 43% formamide, 1% probe and 6% double-distilled water). Hybridization was performed at 50°C o/n in a humid chamber. After SSPE washings at decreasing concentration, endogenous phosphatases were blocked by incubation with a blocking solution (500 μl of 2% BSA, 3 μl of 0.3% Triton X-100 , 500 μl of PBS) for 45 min at RT. The chromogen reaction was developed by incubation with Extravidin-TRITC-conjugated for 2 h at RT in the dark. Slides were counterstained with Hoechst, mounted with an aqueous medium, coverslipped, sealed with nail polish, observed under a fluorescent confocal microscope and images recorded. All slides were processed together, including the right lateral femoral condyle as a negative control.

### Statistical analysis

An analysis of variance was performed on the macroscopic and histological data from the total as well as selected category scores of both groups throughout all time periods. The model of the analysis contained the main effects of treatment and time from surgery and the interaction between them, together with the random effect of the ewe within the period. The Mixed Procedure of SAS 8.2 (SAS Institute Inc., Cary, NC, USA) was used to perform the analysis.

### Availability of supporting data

All the supporting data are included as additional files.

## Additional files

Additional file 1:
**Provided with this submission, shows the semi-quantitative macroscopic and histological scores for the samples’ evaluation and the tables of least square means of macroscopic and histological scores for each time period (1, 2, 6, 12 and 24 months).**
Additional file 2:
**ARRIVE checklist.**

## Abbreviations

ED: Empty defect
ESCs: Embryonic stem cells
ES-like cells: Embryonic stem-like cells
ES: ES-like cells engrafted in the medial femoral condyle defect
FISH: Fluorescent in situ hybridization
GFP: Green fluorescent protein
ICM: Inner cell mass
mAbs: monoclonal antibodies
MSCs: Mesenchymal stem cells
SAT: Sheep SAT 1,114 DNA repeat unit
SRY: Sex-determining region Y-linked gene sequence
