# Supplementary material for: Treatment with embryonic stem-like cells into osteochondral defects in sheep femoral condyles
Source: BMC Vet Res. 2014 Dec 19;10:301. doi: 10.1186/s12917-014-0301-9 (PMC4297431; doi:10.1186/s12917-014-0301-9)
Supplement: Additional file 1: — Provided with this submission, shows the semi-quantitative macroscopic and histological scores for the samples’ evaluation and the tables of least square means of macroscopic and histological scores for each time period (1, 2, 6, 12 and 24 months). [file 12917_2014_301_MOESM1_ESM.docx]

**Table S1.** Semi-quantitative macroscopic score for the samples’ evaluation

*(9 = normal; 0 = worst healing)*

| **Categories** | **Score** | **Qualifications** |
| --- | --- | --- |
| Surface appearance of repairing tissue | 3 | 100 % normal^*^ |
|  | 2 | >75% normal |
|  | 1 | 50-75% normal |
|  | 0 | <50% normal |
| Percent area of defect filled | 3 | 100% |
|  | 2 | >75% |
|  | 1 | 50-75% |
|  | 0 | <50% |
| Graft-recipient tissue integration | 3 | 100% of perimeter |
|  | 2 | >75% |
|  | 1 | 50-75% |
|  | 0 | <50% |

^*^Smooth, white, indistinguishable from normal healthy cartilage.

**Table S2**. Semi-quantitative histological score for the samples’ evaluation.

*(56 = maximum score; 5 = minimum score)*

| **Categories** | | **Grading scale** | **Score** |
| --- | --- | --- | --- |
| Filling of defect |  | Complete | 2 |
|  |  | Partial | 1 |
|  |  | None | 0 |
| Cartilage | Surface regularity | Regular | 2 |
|  |  | Irregular | 1 |
|  | Cell type | Chondroblasts-cytes | 4 |
|  |  | Both chondroblasts and fibroblasts | 3 |
|  |  | Fibroblasts | 2 |
|  |  | Fibroblasts and endothelial cells | 1 |
|  |  | Empty | 0 |
|  | Tissue type | Mature hyaline cartilage | 5 |
|  |  | Immature hyaline cartilage | 4 |
|  |  | Fibrocartilage | 3 |
|  |  | Fibrous tissue | 2 |
|  |  | Granulation tissue | 1 |
|  |  | Empty | 0 |
|  | Continuity between tangential layer proliferation and subchondral ossification | Presence | 1 |
|  |  | Absence | 0 |
| Bone | Ossification | Lamellar bone | 3 |
|  |  | Woven bone | 2 |
|  |  | Subchondral ossification | 1 |
|  |  | Absence | 0 |
|  | Cell Type | Osteogenic cells | 5 |
|  |  | Chondroblasts/cytes | 4 |
|  |  | Both chondroblasts and fibroblasts | 3 |
|  |  | Fibroblasts | 2 |
|  |  | Fibroblasts and endothelial cells | 1 |
|  |  | Empty | 0 |
| Edges of defect | Continuity of articular cartilage edge and filling material | Two edges | 2 |
|  |  | One edge | 1 |
|  |  | None | 0 |
|  | Tidemark | Presence (complete) | 2 |
|  |  | Presence (discontinuous) | 1 |
|  |  | Premature/Absence | 0 |
| Vascularity | Amount | None | 3 |
|  |  | Low | 2 |
|  |  | Medium | 1 |
|  |  | High | 0 |
|  | Distribution | Absence | 5 |
|  |  | Above level of tidemark | 4 |
|  |  | Upper half of defect below tidemark | 3 |
|  |  | Lower half of defect below tidemark | 2 |
|  |  | Entire | 1 |
| Degeneration | Degree of articular deterioration | None | 4 |
|  |  | Mild | 3 |
|  |  | Moderate | 2 |
|  |  | Severe | 1 |
|  | Subchondral bone sclerosis | None or not pertinent | 4 |
|  |  | Mild | 3 |
|  |  | Moderate | 2 |
|  |  | Severe | 1 |
|  | Subchondral bone cysts | None | 4 |
|  |  | Mild | 3 |
|  |  | Moderate | 2 |
|  |  | Severe | 1 |
| Matrix staining | Collagen staining with Azan-Mallory | Marked | 3 |
|  |  | Moderate | 2 |
|  |  | Mild | 1 |
|  |  | Premature | 0 |
|  | Proteoglycan staining with Safranine–O | Marked | 4 |
|  |  | Moderate | 3 |
|  |  | Mild | 2 |
|  |  | Initial production | 1 |
|  |  | Premature/Negative | 0 |
|  | Collagen type II immunostaining | Marked | 3 |
|  |  | Moderate | 2 |
|  |  | Mild | 1 |
|  |  | Premature/Negative | 0 |

**Table S3**. Least square means ± standard error of ES**^*^** and ED**^†^** macroscopic scores for each time period (1, 2, 6, 12 and 24 months)

| **Categories** | **Treatments** | | | | | | | | | |
| --- | --- | --- | --- | --- | --- | --- | --- | --- | --- | --- |
|  | **1 month** | | **2 months** | | **6 months** | | **12 months** | | **24 months** | |
|  | **ES*** | **ED†** | **ES** | **ED** | **ES** | **ED** | **ES** | **ED** | **ES** | **ED** |
| Total macroscopic score | 3.50±1.2 | 1.00±1.2 | 2.33±0.9 | 2.67±0.9 | 4.00±0.9 | 4.00±0.9 | 7.20±0.7 | 6.4±0.7 | 6.25±0.8 | 5.00±0.8 |
| Surface appearance | 1.00±0.5 | 0.00±0.5 | 1.00±0.4 | 1.00±0.4 | 1.33±0.4 | 1.00±0.4 | 2.20±0.3 | 1.80±0.3 | 2.00±0.4 | 1.50±0.4 |
| Filling of defect | 2.50±0.5 | 1.00±0.5 | 1.00±0.4 | 1.00±0.4 | 1.67±0.4 | 1.67±0.4 | 2.80±0.3 | 2.6±0.3 | 2.50±0.4 | 2.00±0.4 |
| Edges integration | 0.00±0.5 | 0.00±0.5 | 0.33±0.4 | 0.67±0.4 | 1.00±0.4 | 1.33±0.4 | 2.20±0.3 | 2.00±0.3 | 1.75±0.3 | 1.50±0.3 |

^*^Embryonic stem-like cells engrafted in the medial femoral condyle; **^†^**Empty defect

**Table S4**. Least square means ± standard error of ES**^*^** and ED**^†^** histological scores for each time period (1, 2, 6, 12 and 24 months)

| **Categories** | **Treatments** | | | | | | | | | |
| --- | --- | --- | --- | --- | --- | --- | --- | --- | --- | --- |
|  | **1 month** | | **2 months** | | **6 months** | | **12 months** | | **24 months** | |
|  | **ES*** | **ED†** | **ES** | **ED** | **ES** | **ED** | **ES** | **ED** | **ES** | **ED** |
| Total histological score | 19.50±3.1 | 16.50±3.1 | 27.33±2.5 | 18.00±2.5 | 39.67±2.5 | 36.00±2.5 | 46.40±2.0 | 40.80±2.0 | 48.75±2.2 | 40.75±2.2 |
| Filling of defect | 1.50±0.3 | 0.50±0.3 | 1.33±0.3 | 0.67±0.3 | 1.67±0.3 | 1.67±0.3 | 2.00±0.2 | 1.80±0.2 | 1.75±0.2 | 1.50±0.2 |
| Cartilage | 3.00±1.2 | 2.00±1.2 | 4.67±1.0 | 0.00±1.0 | 7.00±1.0 | 7.67±1.0 | 9.60±0.7 | 8.60±0.7 | 9.50±0.8 | 7.75±0.8 |
| Matrix | 0.50±1.2 | 0.00±1.2 | 3.00±1.0 | 2.67±1.0 | 8.67±1.0 | 5.67±1.0 | 8.00±0.7 | 7.20±0.7 | 9.00±0.8 | 6.75±0.8 |
| Bone | 1.00±0.6 | 1.00±0.6 | 2.33±0.5 | 0.67±0.5 | 5.00±0.5 | 4.00±0.5 | 7.00±0.4 | 6.60±0.4 | 7.50±0.4 | 7.25±0.4 |
| Edges | 0.00±0.6 | 0.00±0.6 | 1.67±0.5 | 1.33±0.5 | 1.33±0.5 | 1.00±0.5 | 2.80±0.4 | 1.80±0.4 | 3.25±0.4 | 1.50±0.4 |
| Degeneration | 11.00±0.7 | 10.50±0.7 | 11.33±0.5 | 11.00±0.5 | 10.00±0.5 | 11.33±0.5 | 11.20±0.4 | 10.20±0.4 | 10.75±0.5 | 10.00±0.5 |
| Vascularity | 2.50±1.5 | 2.50±1.5 | 3.00±1.2 | 1.67±1.2 | 6.00±1.2 | 4.67±1.2 | 5.80±0.9 | 4.60±0.9 | 7.00±1.0 | 6.00±1.0 |

^*^Embryonic stem-like cells engrafted in the medial femoral condyle; **^†^**Empty defect
