# Supplementary material for: Treatment with embryonic stem-like cells into osteochondral defects in sheep femoral condyles
Source: BMC Vet Res. 2014 Dec 19;10:301. doi: 10.1186/s12917-014-0301-9 (PMC4297431; doi:10.1186/s12917-014-0301-9)
Supplement: Additional file 2: — ARRIVE checklist. [file 12917_2014_301_MOESM2_ESM.pdf]

The ARRIVE guidelines

ITEM		RECOMMENDATION	ANSWERS
Title	1	Provide as accurate and concise a description of the content of the article as possible.	Treatment with embryonic stem-like cells into osteochondral defects in sheep femoral condyles.<br><br>*Susanna Pilichi, Stefano Rocca, Roy R. Pool, Maria Dattena, Gerolamo Masala, Laura Mara, Daniela Sanna, Sara Casu, Maria L. Manunta, Andrea Manunta, Eraldo Sanna Passino*
Abstract	2	Provide an accurate summary of the background, research objectives, including details of the species or strain of animal used, key methods, principal findings and conclusions of the study.	BACKGROUND AND PURPOSE:<br>Defects deriving from trauma or joint disease tend to be repaired with fibrocartilage rather than hyaline cartilage, indeed articular cartilage has a poor intrinsic capacity for regeneration because of its avascularity and very slow cellular turnover. Embryonic Stem cells (ESCs) are very promising, being able to self-renew for prolonged periods without differentiation and to differentiate into tissues from all the 3 germ layers. To date few studies have applied ESCs in cartilage regeneration in animal models and most of them used laboratory animals. This study aimed to evaluate if the local delivery of ES-like cells into osteochondral defects in the femoral condyles of adult sheep may enhance the regeneration process of articular cartilage.<br>EXPERIMENTAL APPROACH:<br>Male Embryonic Stem-like cells (ES) embedded in fibrin glue were engrafted into osteochondral defects in the medial condyles of the left femur in 22 ewes. An identical defect was created in the medial condyle of the controlateral stifle joint and left untreated as a control (empty defect: ED). The ewes were divided into 5 groups. Each groups were euthanized respectively at 1, 2, 6, 12 and 24 months from surgery. New formed tissue was evaluated by macroscopic, histological, immunohistochemical (collagen type II) and fluorescent in situ hybridization (FISH) assays.<br>KEY RESULTS:<br>We observed regenerated tissue in 17 sheep. ES samples had significantly better histologic evidence of repair in ES in respect to controls throughout all considered periods.<br>CONCLUSIONS AND IMPLICATIONS:<br>Histological assessments demonstrated that the local delivery of ES-like cells into osteochondral defects in sheep femoral condyles enhances the regeneration of the articular hyaline cartilage, both in term of architecture and biochemical composition of the matrix.

The ARRIVE guidelines

INTRODUCTION			
Background	3	a. Include sufficient scientific background (including relevant references to previous work) to understand the motivation and context for the study, and explain the experimental approach and rationale.	Articular cartilage has a poor intrinsic capacity for regeneration because of its avascularity and very slow turnover both at the cellular and molecular levels. As consequence, defects occurring as a result of trauma or joint disease tend to be repaired with fibrocartilage rather than hyaline cartilage. With time, degenerative processes frequently occur in the regenerated tissue [1, 2, 3], which may stabilize or progress in relation to 2 main factors: the width and depth of the defect. It has been demonstrated that sheep articular cartilage osteochondral defects 3 mm wide or less re-fill with normal hyaline cartilage, whereas wider defects are replaced by fibrocartilage which, eventually, degenerate in fibrous tissue [4]. This inferior repair tissue is not capable of withstanding the mechanical loads exerted on the tissue during locomotion and the result is eburnation of the subchondral bone [4, 5]. In relation to the depth, superficial defects involving only the articular cartilage do not heal spontaneously [1, 2, 6], while osteochondral defects, penetrating the subchondral bone and thus, gaining access to the mesenchymal stem cells (MSCs) that reside in the bone marrow space, can give rise to regenerated cartilage [7, 8, 9].Surgical treatments used to stimulate cartilage regeneration, in most cases, result only in a delay in the onset of degenerative processes [10, 11, 12, 13, 14 ,15]. Thus, there is a search for alternative solutions and cells engraftment is among the most advanced new technologies in cartilage repair [16, 17, 18, 19, 20]. Considering that in certain degenerative diseases, autologous stem cells are depleted and have reduced proliferative capacity and chondrogenic ability [21, 22], the delivery of heterologous cells may enhance repair or inhibit the progressive loss of joint tissue [21, 22]. Among the several factors to be considered in the choice of the type of cells, there are the ease of harvest, the cell yield and purity and their proliferative and chondrogenic capacity [23]. Autologous Chondrocytes Implantation (ACI), the first technique used to repair focal cartilage defects [24, 25, 26], is associated with donor site morbidity, loss of chondrocyte phenotype upon ex vivo expansion and inferior fibrocartilage formation at the defect site [27, 28, 29]. Thus, new extracorporeal cell sources are sought, mainly stem cells. Among them, Mesenchymal Stem Cells (MSCs) [13, 18, 19, 21, 30], shows the advantages of their immunoevasivity [31] and immunosuppressive effect [32, 33], but they have a limited capacity for self-renewal and proliferation, and differentiation potential impaired with increasing donor age [34]. On the contrary, Embryonic Stem Cells (ESCs) are able to self-renew for prolonged periods without differentiation and, most importantly, to differentiate into a large variety of tissues derived from all 3 germ layers [35, 36, 37, 38].

		b. Explain how and why the animal species and model being used can address the scientific objectives and, where appropriate, the study's relevance to human biology.	The histological appearance of the articular regenerated tissue is essential for the validation of therapeutic interventions [9] and it is likely to be predictive of its functionality and durability [39]. Since in human is possible to perform only small arthroscopic sampling to evaluate the histological aspect of the regenerated tissue, in deep histological studies can be achieved in vivo only in animal models. To date few studies concerning cartilage regeneration have been performed in animal models in vivo [40, 41, 42, 43] and, until recently, most of the research on stem cells has been performed in small laboratory animals [13, 44]. However, they do not represent an optimal model for achieving cartilage regeneration in human. On the contrary, the larger size and weight of adult sheep, which places greater weight-bearing loads on the healing site, as well as the structural, biochemical, physiological and immunological similarities to man and the ease and cheap cost of its management in respect to other species, make sheep an optimal experimental model for future clinical application in human [45, 46].
Objectives	4	Clearly describe the primary and any secondary objectives of the study, or specific hypotheses being tested.	a. Confirm the origin of the stem tissue newly generated by sexing the cells with the PCR in the liquid phase and in in situ hybridization;<br>b. Evaluate the histopathological point of view the reparative process verifying the deposition of type II collagen and comparing the implants with the controls at 1, 2, 6, 12 and 24 months.
METHODS			
Ethical statement	5	Indicate the nature of the ethical review permissions, relevant licences (e.g. Animal [Scientific Procedures] Act 1986), and national or institutional guidelines for the care and use of animals, that cover the research.	The animals were maintained and treated in accordance with Legislative Decree 116/92 guidelines based on European Directive 86/609/EEC. Animal welfare was routinely checked by veterinarians from VTH (Veterinary Teaching Hospital) of the Department of Veterinary Medicine (DVM) and every effort was made to minimize the number of animals used and their suffering. The experimental protocols were approved by the Ethical Committee of the University of Sassari and by the Veterinary control officers of Animal Protection in experimental and clinical studies made in the University of Sassari (Auth. of 06/06/2012).
Study design	6	For each experiment, give brief details of the study design including:<br>a. The number of experimental and control groups.	4 groups of 4 Sarda sheep (1, 2, 6 and 12 month from surgical procedure) and 1 group of 6 Sarda sheep (24 month from surgical procedure) each were studied (total 22 ewes). ES-like cells were engrafted in the osteochondral defect created in the left medial femoral condyle (ES), while the identical defect created in the controlateral stifle joint was left untreated (Empty Defect: ED) and served as a control.

	b. Any steps taken to minimise the effects of subjective bias when allocating animals to treatment (e.g. randomisation procedure) and when assessing results (e.g. if done, describe who was blinded and when).	All animals before the surgery have been adapted to the environmental conditions in the locations of hospitalization at least one month. In this time have been performed rutine analysis for parassites or other disease in progress. After the surgery, all animals were released in paddocks with the same environmental conditions and feeding.<br><br>In the study, n refers to number of animals. From each condyle we got 2 half-lesion (Figure 1. A and B) and from each half lesion we got 4 representative slides for histological staining and immunohistochemistry.
	c. The experimental unit (e.g. a single animal, group or cage of animals).	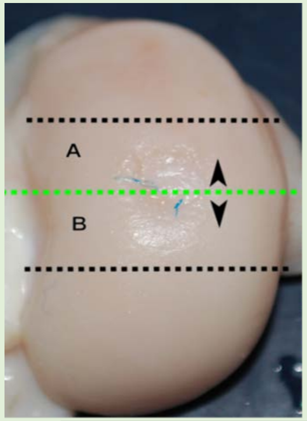 Figure 1 – diagram of dissection of lesion for the histological assay, A upper half lesion and B down half lesion.
	d. A time-line diagram or flow chart can be useful to illustrate how complex study designs were carried out	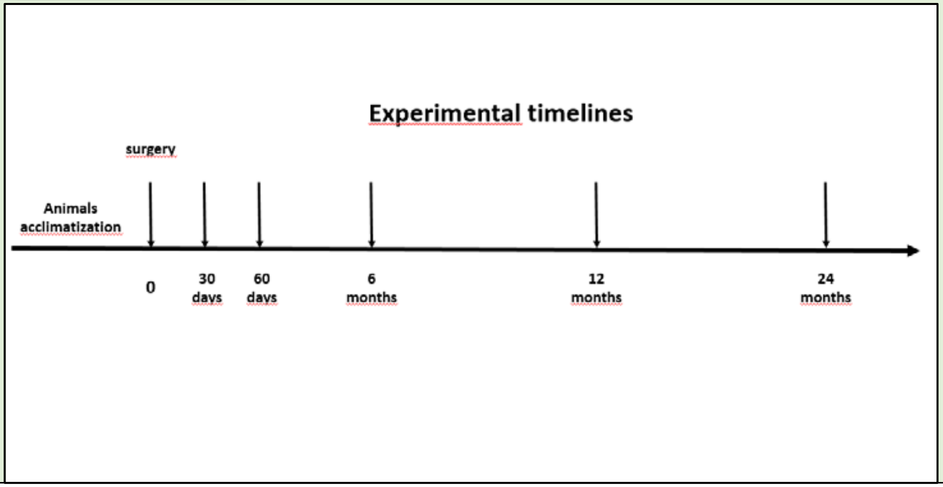

Experimental procedures	7	For each experiment and each experimental group, including controls, provide precise details of all procedures carried out. For example:<br>a. How (e.g. drug formulation and dose, site and route of administration, anaesthesia and analgesia used [including monitoring], surgical procedure, method of euthanasia). Provide details of any specialist equipment used, including supplier(s).	1. Sedation: Diazepam 0.4 mg/kg IV<br>2. Sacro-coccigeal (S4-Co1) epidural anaesthesia: Lidocaine 2 mg/kg<br>3. Induction of the anaesthesia: Ketamine (3 mg/kg IV)<br>4. Maintained of the anaesthesia: Ketamine (3 mg kg-1 h-1) and Fentanyl (8 mcg kg-1 h-1).<br>A lubricated stomach tube of 1 cm of inner diameter was inserted into the rumen to prevent bloating. The animal was positioned in dorsal recumbence in a cradle for thoracic containment, leaving posterior limbs free. With the knee in the maximum flexure, a lateral para-patellar arthrotomy of both stifle joints was performed using a lateral approach and medial patellar dislocation, to expose the articular surface of the weight-bearing area of the medial femoral condyle. A cranio-lateral cutaneous incision, of about 8 cm in length. A full-thickness defect was performed using a punch for chondral biopsy of 6 mm of diameter to mark off the edges of the defect and to cut the cartilage until the calcified portion. Thus, all defects had the same diameter (6 mm) and depth (2 mm) and involved the subchondral bone (Figure 2).During drilling, the area was infused with saline solution to cool the tissue and to avoid dehydration of articular cartilage due to the loss of synovial fluid during the surgical procedure. All animals received an intra-surgical antibiotic and antinflammatory therapy (amoxicillin 25 mg/kg im and ketoprofen 2 mg/kg im), continued for the following post-surgical 4 days, before of the re-positioning of the patella and the suture of the several layers. All surgical procedures were performed by the same operator, in the respect of the welfare laws. Antibiotic and antinflammatory therapy (amoxicillin 40 mg/kg/day im and ketoprofen 2 mg/kg/day im) was administered for 5 days. All animals were kept confined in paddocks for 10 days in groups of 6 animals each, and then were allowed to roam freely on pasture for the rest of the study.<br>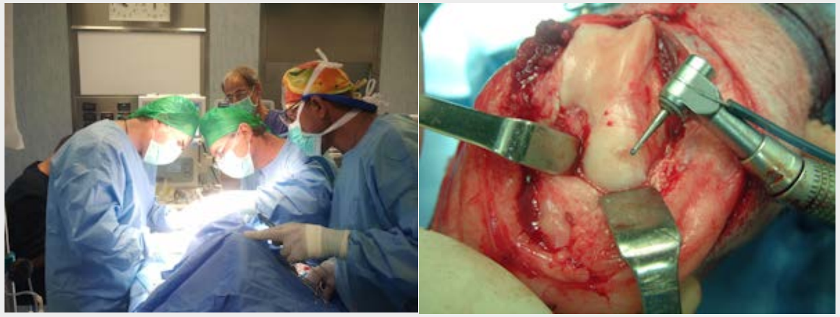<br>Figure 2 – surgery of the knee.

		b. When (e.g. time of day).<br>c. Where (e.g. home cage, laboratory, water maze).	At 9:00 AM<br>Surgical room of Department on Medicine Veterinary of Sassari (EAEVE approved) Figure 3<br>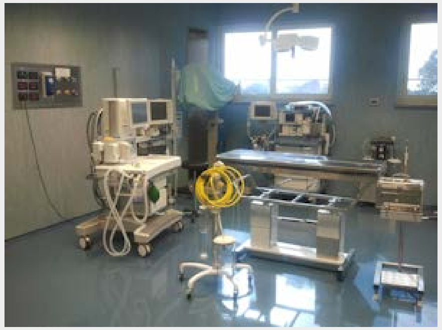<br>Figure 3 – Surgical room
		d. Why (e.g. rationale for choice of specific anaesthetic, route of administration, drug dose used).	This protocol in use provides a complete analgesia and sedation
Experimental animals	8	a. Provide details of the animals used, including species, strain, sex, developmental stage (e.g. mean or median age plus age range) and weight (e.g. mean or median weight plus weight range).<br>b. Provide further relevant information such as the source of animals, international strain nomenclature, genetic modification status (e.g. knock-out or transgenic), genotype, health/immune status, drug or test naïve, previous procedures, etc.	Twenty-two Sarda female ewes, about 5.5 years old and weighing approximately 45 kg, without muscular-skeletal pathologies, were used in this experiment<br>The water was always available and the feed was mixed hay and concentrates
Housing and husbandry	9	Provide details of:<br>a. Housing (type of facility e.g. specific pathogen free [SPF]; type of cage or housing; bedding material; number of cage companions; tank shape and material etc. for fish).	all animals were reared in semi-extensive paddocks (figure 4)<br>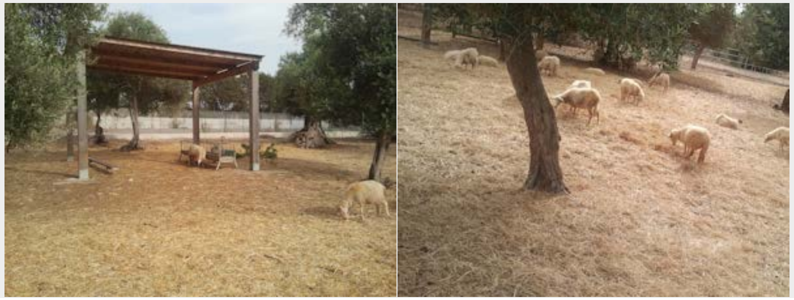<br>Figure 4 semi-extensive paddocks (DVM)

		b. Husbandry conditions (e.g. breeding programme, light/dark cycle, temperature, quality of water etc for fish, type of food, access to food and water, environmental enrichment).<br>c. Welfare-related assessments and interventions that were carried out prior to, during, or after the experiment.	The water was always available and the feed was mixed hay and concentrates<br>See 6b point
Sample size	10	a. Specify the total number of animals used in each experiment, and the number of animals in each experimental group.<br>b. Explain how the number of animals was arrived at. Provide details of any sample size calculation used.<br>c. Indicate the number of independent replications of each experiment, if relevant	22 Sarda female ewes, 4 groups of 4 Sarda sheep (1, 2, 6 and 12 month from surgical procedure) and 1 group of 6 Sarda sheep (24 month from surgical procedure)<br>The experiment wasn’t repeated
Allocating animals to experimental groups	11	a. Give full details of how animals were allocated to experimental groups, including randomisation or matching if done.<br>b. Describe the order in which the animals in the different experimental groups were treated and assessed.	The animals was allocated to treatment groups in order group 1, 2 6, 12 and 24 months<br>The sequence for the analysis of the groups was: group 1, 2, 6, 12 and 24
Experimental outcomes	12	Clearly define the primary and secondary experimental outcomes assessed (e.g. cell death, molecular markers, behavioural changes).	
Statistical methods	13	a. Provide details of the statistical methods used for each analysis.<br>b. Specify the unit of analysis for each dataset (e.g. single animal, group of animals, single neuron).<br>c. Describe any methods used to assess whether the data met the assumptions of the statistical approach.	The Mixed Procedure of SAS 8.2 (SAS Institute Inc., Cary, NC, USA) was used to perform the analysis.<br>An analysis of variance was performed on the macroscopic and histological data from the total and some selected categories scores of both treatments throughout all considered periods. The model of the analysis contained the main effects of treatment and time from surgery and the interaction between them, together with the random effect of the ewe within the period.

<table>
<tr><th colspan="4">RESULTS</th></tr>
<tr><td>Baseline data</td><td>14</td><td>For each experimental group, report relevant characteristics and health status of animals (e.g. weight, microbiological status, and drug or test naïve) prior to treatment or testing. (This information can often be tabulated).</td><td>The animals' health status was monitored throughout the experiments by a health surveillance programme according to Ethical Committee of the University of Sassari and by the Veterinary control officers of Animal Protection in experimental and clinical studies made in the University of Sassari (see point 5)).</td></tr>
<tr><td rowspan="2">Numbers analysed</td><td rowspan="2">15</td><td>a. Report the number of animals in each group included in each analysis. Report absolute numbers (e.g. 10/20, not 50%2).</td><td>ES-like cells were engrafted in the osteochondral defect created in the left medial femoral condyle (ES), while the identical defect created in the controlateral stifle joint was left untreated (Empty Defect: ED) and served as a control. Thus, a total of 44/44 defects were created: 22/44 ES and 22/44 ED (see table 1 of manuscript). Only 17/22 animals were included in the analysis, because 5 sheep died. One sheep belonging to the 24 months-group died at about 12 months post-surgery, and hence it was included with the 12 months-group. Analysis was thus performed on 2/4 sheep in the 1 month-group, 3/4 sheep each in the 2/4 and 6 months-groups, 5/5 sheep in the 12 months-group and 4/6 sheep in the 24 months-group.</td></tr>
<tr><td>b. If any animals or data were not included in the analysis, explain why.</td><td>5/22 animals were not included in the analysis, because 5 sheep died from toxaemic gastroenteritis.</td></tr>
<tr><td>Outcomes and estimation</td><td>16</td><td>Report the results for each analysis carried out, with a measure of precision (e.g. standard error or confidence interval).</td><td>Figure 5 Statistical Analysis of histological grading score

Least square means ± standard error of histological scores compared throughout all considered periods.
<table>
<tr><th>Categories</th><th>Treatments ES*</th><th>ED†</th><th>P value</th></tr>
<tr><td>total histological score</td><td>36.33±1.1^a</td><td>30.41±1.1^b</td><td>^{a,b} < 0.001</td></tr>
<tr><td>filling of defect</td><td>1.65±0.1^a</td><td>1.22±0.1^b</td><td>^{a,b} < 0.05</td></tr>
<tr><td>cartilage</td><td>6.75±0.4^a</td><td>5.20±0.4^b</td><td>^{a,b} < 0.05</td></tr>
<tr><td>matrix</td><td>5.83±0.4^a</td><td>4.46±0.4^b</td><td>^{a,b} < 0.05</td></tr>
<tr><td>bone</td><td>4.57±0.2^a</td><td>3.90±0.2^b</td><td>^{a,b} < 0.05</td></tr>
<tr><td>edges</td><td>1.81±0.2^a</td><td>1.13±0.2^b</td><td>^{a,b} < 0.05</td></tr>
<tr><td>degeneration</td><td>10.86±0.2^a</td><td>10.61±0.2</td><td>n.s.‡</td></tr>
<tr><td>vascularity</td><td>4.86±0.5^a</td><td>3.89±0.5</td><td>n.s.‡</td></tr>
</table>
*Embryonic stem-like cells engrafted in the left medial femoral condyle; †Empty defect created in the right medial femoral condyle. ‡ not significant</td></tr>
<tr><td>Adverse events</td><td>17</td><td>a. Give details of all important adverse events in each experimental group.

b. Describe any modifications to the experimental protocols made to reduce adverse events.</td><td>5 sheep died from toxaemic gastroenteritis, due to the breakage of the fences in a paddock hosting the animals, which, consequently, freely pastured with frost grass, resulting in the onset of the gastro-enteric pathology.
One sheep belonging to the 24 months-group died at about 12 months post-surgery, and hence it was included with the 12 months-group. Analysis was thus performed on 2 sheep in the 1 month-group, 3 sheep each in the 2 and 6 months-groups, 5 sheep in the 12 months-group and 4 sheep in the 24 months-group.</td></tr>
</table>

DISCUSSION			
Interpretation/ scientific implications	18	a. Interpret the results, taking into account the study objectives and hypotheses, current theory and other relevant studies in the literature.	In this study ES showed a significantly better histological healing process as compared to ED in most of the examined categories. In our knowledge, this is the first time that ES-like cells are engrafted in sheep and that engraftments have been evaluated until 24 months from surgery. Up to now, they have been assessed at a maximum period of 18 months in sheep [4], 12 months in goat [21], 8 months in horse [18] and 6 months in laboratory animals [13, 45, 46, 47]. The treatment of ES-like cells, engrafted into osteochondral defects in sheep knee condyles, enhances the regeneration of the articular hyaline cartilage, above all in the long term periods.
		b. Comment on the study limitations including any potential sources of bias, any limitations of the animal model, and the imprecision associated with the results2.	In small ruminants (sheep [4, 20, 44, 49, 50] and goat [5, 7, 21]), most of experiments euthanized animals at 6 months, probably employing the later time period used in laboratory animals. According to the authors: this is a too short period to establish a complete regeneration process in large animals, in agreement with Schneider-Wald [55], who affirms that the follow-up period for assessment of the effectiveness of cartilage regeneration is 12months. According to the authors: this is a too short period to establish a complete regeneration process in large animals, in agreement with Schneider-Wald [55], who affirms that the follow-up period for assessment of the effectiveness of cartilage regeneration is 12months. Moreover, in sheep, several authors [45, 49, 51, 52, 53] perform chondral defect, despite such kind of defect can't heal spontaneously [1, 2, 6]. Even, most of experiments performed in sheep employed surgical techniques without the use of cells [4, 20, 50, 54]. For all these reasons, a comparison of results obtained in this experiment with other trials resulted very difficult to perform.
		c. Describe any implications of your experimental methods or findings for the replacement, refinement or reduction (the 3Rs) of the use of animals in research.	

Generalisability/ translation	19	Comment on whether, and how, the findings of this study are likely to translate to other species or systems, including any relevance to human biology.	Sheep demonstrated to be a valid model for human translational research when compared with laboratory animals, due to the increased stifle size, less effective native cartilage repair and longer lifespan. Moreover, a recent study comparing the differences in geometry and mechanical properties of human, porcine, bovine and ovine articular cartilage, found that human cartilage had a significant largest equilibrium elastic modulus in respect to porcine and bovine cartilage, but not with ovine cartilage [50]. In addition, from the regulatory point of view, the ovine model is one of the suggested large animal models for pre-clinical studies [48].
Funding	20	List all funding sources (including grant number) and the role of the funder(s) in the study.	This work was funded by the MIUR (Ministry of Education, University and Research) in PRIN 2005 call, the title of project was "The use of stem cells in the reparation of cartilage defects. Study on animal model" (L'UTILIZZO DELLE CELLULE STAMINALI NELLA RIPARAZIONE DELLE LESIONI CARTILAGINEE. STUDIO SU MODELLO ANIMALE"). The funders had no role in study design, data collection and analysis, decision to publish, or preparation of the manuscript.

REFERENCE

1. Grassel S, Ahmed N: Influence of cellular microenvironment and paracrine signals on chondrogenic differentiation. Front Biosci 2007;12:4946-4956.
2. Steinert AF, Ghivizzani SC, Rethwilm A, Tuan RS, Evans CH, Nöth U: Major biological obstacles for persistent cell-based regeneration of articular cartilage. Arthritis Research & Therapy 2007 9:213-228.
3. Frenkel S, Di Cesare P: Degradation and repair of articular cartilage. Front Biosci 1999;4:671-685.
4. Akens MK, von Rechenberg B, Bittmann P, Nadler D, Zlinszky K, Auer JA: Long-term in vivo studies of photo-oxidized osteochondral transplant in sheep. BMC Muskoloskel Disord 2001;2:9-20..
5. Butnariu-Ephrat M, Robinson D, Mendes DG, Halperin N, Nevo Z: Resurfacing of goat articular cartilage by chondrocytes derived from bone marrow. Clin Orthop 1996;330:234-243.
6. Mano JF, Reis RL: Osteochondral defects: present situation and tissue engineering approaches. J Tissue Engin Reg Med 2007;1:261-273.
7. Jackson DW, Scheer MJ, Simon TM: Cartilage substitutes: overview of basic science and treatment options. J Am Acad Orthop Surg 2001;9(Suppl 1):37-52.
8. Buckwalter JA, Mankin HJ: Articular cartilage repair and transplantation. Arthritis Rheum 1998;41:1331-1342.
9. Shapiro F, Koide S, Glimcher MJ: Cell origin and differentiation in the repair of full-thickness defects of articular cartilage. J Bone Joint Surg Am 1993;75:532-553.
10. Friedman M, Berasi C, Fox J, Del Pizzo W, Snyder S, Ferkel R: Preliminary results with abrasion arthroplasty in the osteoarthritic knee. Clin Orthop 1984;82:200-205,
11. Gill TJ, Asnis PD, Berkson EM: The treatment of articular cartilage using the microfracture technique. J Orthop Sports Phys Ther 2006;36 (10):728-738,
12. Insall J: The Pridie debridement operation for osteoarthritis of the knee. Clin Orthop Relat Res 1974;101:61-7,

13. Wakitani S, Goto T, Pineda SJ, Young RG, Mansour JM, Caplan AI, Goldberg VM: Mesenchymal cell-based repair of large, full-thickness defects of articular cartilage. J Bone Joint Surg 1994;76:579-592.

14. Wakitani S, Aoki H, Harada Y, Sonobe M, Morita Y, Mu Y, Tomita N, Nakamura Y, Takeda S, Watanabe TK, Tanigami A: Embryonic stem cell form articular cartilage, not teratomas, in osteochondral defects of rat joints. Cell Transpl 2004;13:331-336

15. Martinez-Carranza N, Berg HE, Hultenby K, Nurmi-Sandh H, Ryd L, Lagerstedt AS: Focal knee resurfacing and effects of surgical precision on opposing cartilage. A pilot study on 12 sheep. Osteoarthritis Cartilage 2013;21(5):739-45.

16. Dattena M, Pilichi S, Rocca S, Mara L, Casu S, Masala G, Manunta L, Manunta A, Passino ES, Pool RR, Cappai P: Sheep embryonic stem-like cells transplanted in full-thickness cartilage defects. J Tissue Eng Regen Med 2009;3:175-187.

17. Matzumoto T, Okabe T, Ikawa, Miosge N, Hartmann M, Maelicke C, Herken R: Expression of collagen type I and type II in consecutive stages of human osteoarthritis. Histochem Cell Biol 2004;122:229-236.

18. Wilke MM, Daryl VN, Nixon AJ: Enhanced early chondrogenesis in articular defects following arthroscopic mesenchymal stem cell implantation in an equine model. J Orthop Res 2007;25(Suppl 7):913-925.

19. Undale AH, Westendorf JJ, Yaszemski MJ, Khosla S: Mesenchymal stem cells for bone repair and metabolic bone diseases. Mayo Clin Proc 2009;84 (Suppl 10):893-902.

20. Solter D, Knowles BB: Monoclonal antibody defining a stage-specific mouse embryonic antigen (SSEA-1). Proc Natl Acad Sci U S A. 1978,75(11):5565-9.

21. Murphy JM, Fink DJ, Hunziker EB, Barry FP: Stem cell therapy in a caprine model of osteoarthritis. Arthritis Rheum 2003;48:3464-3474.

22. Murphy JM, Dixon K, Beck S, Fabian D, Feldman A, Barry F: Reduced chondrogenic and adipogenic activity of mesenchymal stem cells from patients with advanced osteoarthritis. Arthritis Rheum 2002;46:704-713.

23. van Osch GJVM, Brittberg M, Dennis JE, Bastiaansen-Jenniskens YM, Erben RG, Konttinen YT, Luyten FP: Cartilage repair: past and future - lessons for regenerative medicine. J. Cell. Mol. Med 2009; 13(Suppl. 5):792-810.

24. Brittberg M, Lindahl A, Nilsson A, Ohlsson C, Isaksson O, Peterson L: Treatment of deep cartilage defects in the knee with autologous chondrocyte transplantation. New Engl J of Med 1994; 331(14):889-895.

25. Gillogly SD, Myers TH, Reinold MM. Treatment of large full-thickness chondral defects of the knee with autologous chondrocyte implantation. Arthroscopy 2003;19:147-53.

26. Minas T, Chiu R: Autologous chondrocyte implantation. Am J Knee Surg 2000;13:41-50.

27. Toh WS, Lee EH, Cao T: Potential of Human Embryonic Stem Cells in Cartilage Tissue Engineering and Regenerative Medicine. Stem Cell Rev and Rep 2011;7:544-559.

28. Schnabel M, Marlovits S, Eckhoff G, Fichtel I, Gotzen L, Vécsei V, Schlegel J: Dedifferentiation-associated changes in morphology and gene expression in primary human articular chondrocytes in cell culture. Osteoarthritis and Cartilage 2002; 10(1):62-70.

29. von der Mark K, Gauss V, von der Mark H, Müller P: Relationship between cell shape and type of collagen synthesized as chondrocytes lose their cartilage phenotype in culture. Nature 1977;267(5611):531-532.

30. Matzumoto T, Okabe T, Ikawa T, Iida T, Yasuda H, Nakamura H, Wakitani S: Articular cartilage repair with autologous bone marrow mesenchymal cells. J Cell Physiol 2010;225(Suppl2):291-295.

31. Javazon EH, Beggs KJ, Flake AW: Mesenchymal stem cells: paradoxes of passaging. Exp Hematol 2004; 32(5):414-425.

32. Di Nicola M, Carlo-Stella C, Magni M, Milanesi M, Longoni PD, Matteucci P, Grisanti S, Gianni AM: Human bone marrow stromal cells suppress T-lymphocyte proliferation induced by cellular or non –specific mitogenic stimuli. Blood 2002;99(10):3838-3843.

33. Bartholomew A, Sturgeon C, Siatskas M, Ferrer K, McIntosh K, Patil S, Hardy W, Devine S, Ucker D, Deans R, Moseley A, Hoffman R: Mesenchymal stem cells suppress lymphocyte proliferation in vitro and prolong skin graft survival in vivo. Exp Hematol 2002;30(1):42-48.

34. Payne KA, Didiano DM, Chu CR: Donor sex and age influence the chondrogenic potential of human femoral bone marrow stem cells. Osteoarthritis and Cartilage 2010;18(5),705-713.

35. Thomson JA, Itskovitz-Eldor J, Shapiro SS, Waknitz MA, Swiergiel JJ, Marshall VS, Jones JM: Embryonic stem cell lines derived from human blastocysts. Science 1998;282(5391):1145–1147.

36. Hwang NS, Varghese S, Elisseeff J: Derivation of chondrogenically-committed cells from human embryonic cells for cartilage tissue regeneration. PLoS One 2008; 3(6):e2498.

37. Toh WS, Lee EH, Guo XM, Chan JK, Yeow CH, Choo AB, Cao T: Cartilage repair using hyaluronan hydrogel-encapsulated human embryonic stem cell-derived chondrogenic cells. Biomaterials 2010;31(27):6968-6980.

38. Toh WS, Yang Z, Liu H, Heng BC, Lee EH, Cao T: Effects of culture conditions and bone morphogenetic protein 2 on extent of chondrogenesis from human embryonic stem cells. Stem Cells 2007;25(4):950-960.

39. Mainil-Varlet P, Aigner T, Brittberg M et al. Histological assessment of cartilage repair: a report by the Histology endpoint Committee of the International Cartilage Repair Society. J Bone Joint Surg Am 2003;85:45-57

40. Dattena M, Mara L, Alì A, Cappai P: Lambing rate of vitrified blastocysts is improved by embryo culture with BSA and hyaluronan. Mol Reprod Dev 2007;74(Suppl 1):42-47.

41. Sanna E, Sanna MP, Loddo C, Sanna L, Mura M, Cadelano T, Leoni A: Endogenous jaagsiekte sheep retrovirus RNA is expressed by different cell types in ovine fetus and placenta. Eur J Histochem 2002;46(Suppl 3):273–280.

42. Buckwalter JA, Mankin HJ: Articular cartilage. Part I: tissue design and chondrocyte–matrix interactions. Instr Course Lect. 1998;47:477-86.

43. Wakitani S, Kimura T, Hirooka A, Ochi T, Yoneda M, Yasui N, Owaki H, Ono K: Repair of rabbit articular surfaces with allograft chondrocytes embedded in collagen gels. J Bone Joint Surg 1989;71(1):74-80.

44. Vasara AI, Hyttinen MM, Lammi MJ, et al.; Subchondral bone reaction associated with chondral defect and attempted cartilage repair in goats. Calcif Tissue Int 2004;74: 107–114.

45. Mrugala D, Bony C, Neves N, et al.; Phenotypic and functional characterization of ovine mesenchymal stem cells: application to a cartilage defect model. Ann Rheum Dis 2008;67: 288–295.

46. Jiang CC, Chiang H, Liao CJ, Lin YJ, Kuo TF, Shieh CS, Huang YY, Tuan RS: Repair of porcine articular cartilage defect with a biphasic osteochondral composite. J Orthop Res. 2007;25(10):1277-90.

47. Hunziker EB, Kapfinger E: Removal of proteoglycans from the surface of defects in articular cartilage transiently enhances coverage by repair cells. J Bone Joint Surg Br. 1998;80(1):144-50.

48. 48 Endres M, Neumann K, Zhou B, Freymann U, Pretzel D, Stoffel M, Kinne RW, Kaps C. An ovine in vitro model for chondrocyte-based scaffold-assisted cartilage grafts. J Orthop Surg Res. 2012;7:37.

49. Ivkovic A, Pascher A, Hudetz D, Maticic D, Jelic M, Dickinson S, Loparic M, Haspl M, Windhager R, Pecina M: Articular cartilage repair by genetically modified bone marrow aspirate in sheep. Gene Ther 2010;17(6):779-89.

50. von Rechenberg B, Akens MK, Nadler D, Bittmann P, Zlinszky K, Kästner SBR, Auer JA: Mosaicplasty with photooxidized, mushroom-shaped, bovine, osteochondral xenografts in experimental sheep. Vet Comp Orthop Traumatol 2006;3:147-156

51. Caminal M, Moll X, Codina D, Rabanal RM, Morist A, Barrachina J, Garcia F, Pla A, Vives J, Transitory improvement of articular cartilage characteristics after implantation of polylactide:polyglycolic acid (PLGA) scaffolds seeded with autologous mesenchymal stromal cells in a sheep model of critical-sized chondral defect. Biotechnol Lett. 2014;36(10):2143-53.

52. Power J, Hernandez P, Guehring H, Getgood A, Henson F, Intra-articular injection of rhFGF-18 improves the healing in microfracture treated chondral defects in an ovine model. J Orthop Res. 2014 May;32(5):669-76.

53. Seedhom BB, Luo ZJ, Goldsmith AJ, Toyoda T, Lorrison JC, Guardamagna L, In-situ engineering of cartilage repair: a pre-clinical in-vivo exploration of a novel system. Proc Inst Mech Eng H. 2007 Jul;221(5):475-88.

54. von Rechenberg B, Akens MK, Nadler D, Bittmann P, Zlinszky K, Neges K, Auer JA: The use of photooxidized, mushroom-structured osteochondral grafts for cartilage resurfacing – a comparison to photooxidized cylindrical grafts in an experimental study in sheep. Osteoarthritis and Cartilage 2004; 12:201-216

55. Schneider-Wald B, von Thaden AK, Schwarz ML: Defect models for the regeneration of articular cartilage in large animals. Orthopade: 2013;Apr;42(4):242-53.
